# Racial concentration and dynamics of COVID-19 vaccination in the United States

**DOI:** 10.1016/j.ssmph.2022.101198

**Published:** 2022-08-18

**Authors:** Cary Wu

**Affiliations:** Department of Sociology, York University, 4700 Keele Street, Toronto, Ontario, M3J 1P3, Canada

## Abstract

This article considers how county-level concentrations of Asians, Blacks, Hispanics, and Whites are associated with COVID-19 vaccination differently. I argue that racially specific mechanisms-differential concentrations of social vulnerability and political ideology by race-are likely to create diverse associations between racial concentration and COVID-19 vaccination not only across racial groups but also within racial groups over time from early rollout to the time after COVID-19 vaccines became widely available. I test this argument by drawing on data from multiple sources that include county-level information on COVID-19 vaccination rates, racial population make-ups, and measures of political ideology and community vulnerability. Results show that the association between racial concentration and COVID-19 vaccination changes substantially across and within racial groups over time. Counties with higher percent of Asians and percent of Whites have higher vaccination rates at earlier time intervals whereas counties with higher percent of Latinos and percent of Blacks show lower vaccination rates. This trend flips at later dates for percent of Blacks, percent of Latinos, and percent of Whites. Results from multilevel regression models and mediation analysis controlling for vaccine hesitancy show that social vulnerability and political ideology are the underlying factors and their differential associations with diverse racial concentrations help create the racially specific and time-varying patterns.

## Highlights

• Racial concentration and COVID-19 vaccination are associated differently across race groups.
• Racial concentration and COVID-19 vaccination are associated differently within race groups over time .
• Social vulnerability and political dynamics are the underlying factors for the racially specific and time varying patterns.

The United States began COVID-19 vaccinations on December 14, 2020. Since COVID-19 vaccines became available, they have been powerful tools to control the spread of the virus and help prevent serious illness and death for infected individuals (Kim, 2021; Pilishvili et al., 2021). However, the benefits have not been equally shared. Data from the Centers for Disease Control and Prevention (CDC) show that COVID-19 vaccination rates vary substantially and rise unevenly over time as COVID-19 waves continue across both geographic areas as well as socio-demographic groups (Centers for Disease Control and Prevention, 2022; see also Alaran et al., 2021).

Race has been a consistent predictor of COVID-19 vaccination rates. Two patterns are well documented. First, vaccination rates have been much lower among Blacks and Hispanics as compared to Asians and Whites (Ndugga et al., 2021). This is especially concerning given that racial minorities, Blacks and Hispanics in particular, are at a higher risk of infections and becoming sicker and dying more often from COVID-19 (Berkowitz et al., 2021; Millett et al., 2020; McFadden et al., 2021; Nguyen et al., 2022; DiRago, 2022). Second, communities that are disproportionately populated by racial minorities, again Blacks and Hispanics in particular, report significantly lower vaccination rates (Anderson & Ray-Warren, 2022; Brown, Young, & Pro, 2021; Hughes et al., 2021). The same communities also experience higher COVID-19 infections and deaths (Berkowitz et al., 2021; Bibbins-Domingo, 2020; Gaynor & Wilson, 2020; Godoy & Wood, 2020; Tan et al., 2022). For this reason, there have been growing discussions on factors that may lead to the lower vaccine uptake among racial and ethnic minorities.

Largely, current discussions have focused on finding “issues” among racial and ethnic minorities, particularly pointing to their higher levels of vaccine hesitancy. Racial and ethnic minorities are often socioeconomically disadvantaged and their higher vulnerability to myths and misinformation, greater perceived barriers to obtaining COVID-19 vaccines, and higher concern about COVID-19 cost and safety could make them more hesitant toward vaccines (Ruiz & Bell, 2021; Khubchandani & Macias, 2021; Momplaisir et al., 2021; Bateman et al., 2022). Their vaccine hesitancy could also come from their lower trust in science and medical establishments due to historical and ongoing discrimination and racial injustices including unconsented and unethical medical experimentation and research practices such as the Tuskegee Syphilis experiment (Cardona et al., 2021; Kricorian et al., 2021; McFadden et al., 2021).

Still, vaccine hesitancy should not be taken as the main explanation for why vaccination rates are lower among racial minorities. In fact, vaccine acceptance is not always lower among racial minorities (Hooper et al., 2021; Kelly et al., 2021). On the contrary, survey data show that Asian Americans, for example, are the group most willing to get themselves vaccinated among all Americans (Funk & Tyson, 2020). Blacks have also become more willing to get vaccinated over time (Agarwal et al., 2021). Further, more recent research suggests that vaccine hesitancy is not the root cause for why the lower vaccine uptake among racial minorities. Indeed, the overall vaccine hesitancy cannot explain the racial disparities in vaccine uptake in a county (Agarwal et al., 2021; Hooper et al., 2021). Real-time data from the CDC show that the actual vaccination rates (received at least one dose) have become higher among Asians as compared to Whites since April 2021 when COVID-19 vaccines become widely available to everyone. Since January 2022, Latinos have also shown higher rates than Whites. As of April 2022, Blacks still show lower vaccination rates, but the differences between Blacks and Whites have narrowed over time (Ndugga et al., 2021).

These changing patterns suggest three important points about how race and COVID-19 vaccination may be related. First, focusing too much on vaccine hesitancy could fall into the victim-blaming trap. It overlooks the need for public health systems to become more trustworthy and accessible as well as the structural barriers that underlie how and why race is a consistent predictor of vaccination uptake (Corbie-Smith, 2021). Second, racial minorities cannot be simply reduced to as one minority group in researching the race and COVID-19 vaccination association. There are substantial disparities in COVID-19 vaccination rates across racial groups that could be related to racially specific group experiences and barriers. Third, time matters. The fact that, over time, COVID-19 vaccination rates have risen unevenly across racial groups suggests that mechanisms underlying the racially differential distributions of COVID-19 vaccination can also be time dependent.

To challenge the public narrative that foregrounds vaccine hesitancy as the root cause for the lower vaccination rates among racial and ethnic minorities, in this article I go beyond the individual-level race and COVID-19 vaccination association and consider how residential concentration of racial and ethnic populations is associated with COVID-19 vaccine uptake. Exploring how racial concentration affects COVID-19 vaccination at the place level provides a way to test how the structural dynamics that connect race to inequitable access to COVID-19 vaccines. This is especially needed as large evidence shows that residential areas with higher concentrations of racial minorities have disproportionately experienced more COVID-19 infections and deaths (Gaynor & Wilson, 2020; Tan et al., 2022; Berkowitz et al., 2021). To this point, Khanijahani and Tomassoni (2022:368) emphasize that “the spatialized nature of structural and environmental racism and socioeconomic disadvantage, along with the infectious nature of COVID-19, calls for attention beyond the individual characteristics.” Hence, establishing a connection between residential concentration of racial and ethnic minorities and COVID-19 inequalities beyond the individual-level helps defuse the “victim-blaming” narratives about why racial and ethnic groups are hardest hit by COVID-19 (see also Tan et al. 2022).

Existing studies have already shown the connection between racial concentration and COVID-19 vaccination, but they tend to focus on a specific racial group, for example, Black concentration (e.g., Agarwal et al., 2021; Millett et al., 2020; Khanijahani and Tomassoni 2022; Gaynor & Wilson, 2020; Anderson & Ray-Warren, 2022) or have grouped all non-white minorities as one group under the concept of minority concentration (e.g., Yang et al., 2021). In particular, few studies have considered the racially-specific mechanisms underlying the effects of racial concentration across race categories. This is important given not only can the association between racial concentration and COVID-19 vaccination vary in patterns across race categories, but the mechanisms underlying the associations could also be different. In this article, I separate county-level residential concentrations by diverse race categories including concentrations of Asians, Blacks, Hispanics, and Whites and consider how they affect COVID-19 vaccine uptake differently. I argue that racially specific mechanisms-differential concentrations of social vulnerability and political ideology by race-are likely to create diverse associations between racial concentration and COVID-19 vaccination not only across racial groups but also within racial groups over time. Specifically, I test how the two widely identified factors that connect racial concentration with COVID-19 vaccination, namely, structural vulnerability and political ideology (Agarwal et al., 2021; Brown, Young, et al., 2021; Hughes et al., 2021), may play differential roles in explaining the impacts of racial concentrations on COVID-19 vaccination across race categories and within race categories over time.

Furthermore, I consider the time dimension of the race and COVID-19 vaccination association. The supply of COVID-19 vaccines as well as narratives and scientific evidence surrounding COVID-19 vaccines have been changing over the course of the pandemic. These changes are likely to affect the effects of race and racial concentration on COVID-19 vaccination. COVID-19 vaccines became available on December 14, 2020, but not to everyone during the early rollout period. Only until the end of April 2021, did vaccines become widely available to everyone who wants a shot. Hence, access to COVID-19 vaccines and therefore racial disparities in vaccination rates could be more related to socioeconomic advantages or disadvantages during early rollout due to the limited supply of COVID-19 vaccines than later as the availability of vaccines increases. In fact, existing research shows that vaccination rates during March and April 2021 were negatively associated with poverty and the uninsured population even though vaccines have been free to everyone regardless of health insurance coverage (DiRago, Li, et al., 2022). For example, both vaccination rates and the increases in vaccination were found to be lower among socioeconomically disadvantaged Black and Hispanic communities than in more affluent, Asian, and White communities (Anderson & Ray-Warren, 2022; DiRago, Li, et al., 2022). The increased variability of vaccines over time, however, could change the dynamics.

## 1 Racial concentration and COVID-19 vaccination

In the United States, as in many other places, residential areas are strongly and increasingly segregated by race (Logan & Schneider, 1984; Massey et al., 2009), leading to the clustering of certain racial groups in different geographic areas (Lichter, 1985; Wright, Ellis, & Holloway, 2014, pp. 111–134). The clustering of different racial and ethnic populations is not only associated with concentrations of different ethnic cultures and activities (e.g., food and restaurants), but also concentrations of advantages/disadvantages as well as different political dynamics. Areas that are disproportionately populated by racial minorities are often those with higher concentrations of socioeconomic disadvantages, heighten social problems including violence and crime, and poorer population health (Sampson et al., 1997; McLaughlin & Stokes, 2002; Quillian, 2012; see also Wu, 2020). In fact, racial concentration and therefore racial segregation leading to the spatial concentrations of advantage and disadvantage is widely considered to be the primary processes in the creation of durable racial inequalities in the United States (Massey, 2009; Sampson & Wilson, 2020; White & Borrell, 2011; Wilson, 1987). Although racial concentration and racial segregation are two distinct concepts, they are related in important ways. On one hand, racial concentration, the process of people of different racial groups moving into different geographical areas, creates patterns of racial segregation (Logan & Schneider, 1984). On the other hand, racial segregation is the central mechanism underlying how racial concentration is associated with the concentration of disadvantages. Residential segregation often increases the concentration of disadvantages for racial minorities, but not for Whites (Krivo et al., 1998; Peterson & Krivo, 1999).

County-level racial concentration has also been the central mechanism underlying how Americans from different racial and ethnic groups have fared differently during the COVID-19 pandemic. Growing research has documented that areas with higher concentrations of racial and ethnic minorities have experienced more negative impacts of the COVID-19 pandemic including higher numbers of infections and deaths due to the concentrations of social disadvantages such as higher rates of poverty, lower social cohesion, and poorer living conditions as well as limited access to resources such as healthy food availability, engagement in physical activity, clean air and water, social capital, and healthcare (e.g., Ahmad et al., 2020; Berkowitz et al., 2021; Brown, Lewis, & Davis, 2021; Gaynor & Wilson, 2020; Godoy & Wood, 2020; Makridis & Wu, 2021; Strully et al., 2021; Tan et al., 2022). These disadvantaged communities are less resilient in their ability to respond to and recover from the waves of the pandemic because they are in short supply of vital resources to contend with the spread of the virus such as sanitizers, masks, as well as testing facilities and health care resources (Godoy & Wood, 2020; Gaynor & Wilson, 2020). These communities also show lower COVID-19 vaccination rates (DiRago, Li, et al., 2022; Hughes et al., 2021; Khubchandani & Macias, 2021; McFadden et al., 2021), and this exacerbates the unequal impacts of the pandemic. In Maryland, for example, Cardona et al. (2021) show that counties with higher concentrations of Blacks and Latinos have higher infection, morbidity and mortality from COVID-19, but lower vaccination rates as compared to counties with predominantly Whites. The same pattern has also been documented nationwide (Agarwal et al., 2021; Brown, Young et al., 2021; Hughes et al., 2021).

Two essential structural factors could underlie why COVID-19 vaccination rates are lower among communities that are populated with racial and ethnic minorities. First, racial concentration is associated with the concentrations of social vulnerabilities. Research has widely established that higher concentrations of racial and ethnic minorities often mean higher concentrations of disadvantages and vulnerabilities that are detrimental to population health including, for example, higher rates of poverty, lower social cohesion, and poor housing and crowded living conditions as well as higher barriers to accessing resources such as healthy food, clean air and water, and health care facilities and resources (e.g., McLaughlin & Stokes, 2002). This is in line with the fundamental cause theory that suggests socioeconomic inequality and structural racism as the root causes of racial and ethnic disparities in health outcomes (Link & Jo, 1995; Phelan & Link, 2015; Williams & Collins, 2016). Socioeconomic disadvantages and structural racism could help explain the association between racial concentration and COVID-19 vaccination through both community and structural vulnerabilities. Members of disadvantaged communities are more likely to experience poor health, and they could be significantly more threatened by COVID-19 (Vargas et al., 2021) and show greater safety concerns about getting vaccinated (Hughes et al., 2021). Disadvantaged and racial minorities are also not receiving proportionate allocations for COVID-19 vaccines and experience higher barriers to accessing vaccines (Hughes et al., 2021; Khubchandani & Macias, 2021). Existing research shows, for example, a higher degree of racial-ethnic minority clustering is associated with fewer vaccination sites and fewer vaccine doses due to the lack of hospitals and physicians’ offices in these areas (Anderson & Ray-Warren, 2022).

Second, racial concentration could be associated with COVID-19 vaccination through concentrations of differential political cultures. Growing research suggests political ideology plays a distinctive role in the domain of public health (Bilewicz & Soral, 2021). Political ideology becomes influential in shaping people's views and behaviors because, when information is lacking in times of crisis, it provides people with a readily available framework for making sense of what is going on (MacKendrick, 2018; Swidler, 1986; Vargas et al., 2021). Political ideology is also associated with a wide range of important factors such as trust in government, beliefs in science, as well as beliefs about how the healthcare system should be structured, all of which can yield significant implications for how people make sense of public health issues (Nisbet et al., 2015; Gelman et al., 2010, April). For example, compared to Democrats, Republicans are also more likely to see healthcare and well-being as an individual, rather than a state, responsibility (Henderson & Hillygus, 2011). Individuals with less trust in government are less likely to comply with COVID-19 control measures such as mask wearing and social distancing (Wu, 2021). When it comes to the COVID-19 vaccination, research has shown that communities with a high percentage of Republican voters (or 2020 Trump vote share) have lower vaccination rates (Albrecht et al., 2022; Sun & Monnat, 2021). Political conservatism has become increasingly associated with skepticism toward science and vaccines (Evans & Feng, 2013; Gauchat, 2012, 2015), and during the pandemic, greater skepticism toward COVID-19 and COVID-19 vaccines (Cowan et al., 2021, Cowan et al., 2021; Diamond, 2021, p. 20; Evans & Hargittai, 2020; Scheitle and Corcoran, 2021, Scheitle and Corcoran, 2021). Differential exposure to media channels and social networks could explain the observed asymmetric polarization between self-identified Democrats and Republicans. For example, exposure to Trump's anti-vaccination tweets could shift the public's sentiment regarding vaccination (Hornsey, 2020). Substantial variations in political ideologies among Americans from different racial and ethnic groups can therefore create different political dynamics across places at an aggregate level, which could lead to racially specific patterns when studying the association between racial concentration and vaccination at the place level.

## 2 This study: racially-specific and time-varying patterns

This study advances current research in two major ways. First, the vast majority of current research on the association between racial concentration and COVID-19 vaccination has failed to consider racially specific mechanisms underlying how racial concentration and COVID-19 vaccination are related across race categories. Concentrations of different racial and ethnic populations are associated with differential concentrations of both social vulnerability and political dynamics. Not only are members of different racial and ethnic groups experience socioeconomic disadvantages at different levels, but their political views and ideologies are also not uniform. For example, Asian concentration is often associated with concentrations of advantages and higher levels of socioeconomic status. This is because Asian Americans often show higher median household incomes, lower poverty rates and a higher proportion of college educated or more, although there is wide variation across Asian subgroups (Budiman, Cilluffo, & Ruiz, 2019). Further, Asians exhibit lower levels of residential segregation than both Hispanics and Blacks despite they still show moderate levels of segregation from Whites (Wilkes & Iceland, 2004; Logan, 2013; Logan & Zhang, 2010). In contrast, Black concentration is more likely to be associated with concentrations of poor health and substantial health disparities (McLaughlin & Stokes, 2002; Williams & Collins, 2016). Concentrations of different racial populations also mean concentrations of different political ideologies. The majority of Black Americans identify as Democrats, and Asians and Hispanics are more likely than Blacks to identify as Republicans (Herrick, 2016). Hence, there is a need to consider racially diverse patterns and mechanisms in exploring how concentrations of racial and ethnic populations and COVID-19 vaccinations are related.

Second, no current studies have considered the changing dynamics of racial concentrations and the underlying factors that shape COVID-19 vaccination over time. I consider the time dimension of the association between racial concentration and COVID-19 vaccination rates. Indeed, factors including both social vulnerability and political ideology that underlie the association between racial concentrations and COVID-19 vaccinations may change over the course of the COVID-19 vaccines rollout. Specifically, I would expect that access to COVID-19 vaccines is associated with racial concentration of social vulnerability more strongly during early rollout when the supply of COVID-19 vaccines was limited. As the availability of vaccines increases, this may change. After vaccines became widely available, socioeconomic advantage may play a lesser role in shaping the distribution of COVID-19 vaccines. Instead, political ideology may play an increasing role in shaping how racial concentrations affect the COVID-19 vaccination rates. Since April 2021, COVID-19 vaccines have become widely available, and they are free to everyone. Therefore, political ideology that shapes how people think of vaccines and public health issues may matter differently in changing contexts.

Taken together, the goal of this study is two-fold. First, I seek to demonstrate the association between racial concentration and COVID-19 vaccination changes in patterns across concentrations of Asians, Blacks, Hispanics, and Whites as well as within racial groups over time from early rollout to the time after COVID-19 vaccines became widely available to everyone. Second, I test the unequal roles social vulnerability and political ideology play in the association between racial concentration and COVID-19 vaccination across race categories and over time, creating the racially specific and time-varying patterns.

## 3 Data and measures

### 3.1 Data

Data for this study come from multiple sources. The main outcome variables are the county-level COVID-19 vaccination rates, which can be retrieved from the Centers for Disease Control and Prevention (CDC). The data include historical and real-time rates of populations who are fully vaccinated (received second dose of a two-dose vaccine or one dose of a single-dose vaccine) as well as the percent of population who received at least one COVID-19 vaccine dose and who received a booster dose across almost all US counties. These rates are also separated by age groups, but the focus of this study is on rates for adults aged 18+ across since the rollout only expanded to children at a much later time (e.g., May 2021 for children ages 12 to 15).

To demonstrate the changing dynamics over time, I selected the rates at four different times, namely, January 2021, April 2021, as well as one year after in January 2022, and the most recent rates in April 2022, although the CDC has been updating the data on vaccination rates almost daily since the United States began COVID-19 vaccinations on December 14, 2020. Specifics about the data on county-level rates can be found on the CDC website (Centers for Disease of Control and Prevention 2022). I focus only on these four time points to simplify the analysis and facilitate easy interpretations of the results. I chose the rate by the end of January 2021 to indicate the early rollout period when the supply of vaccines was limited. There is also little point to focus on rates before January 2021 since most counties had very low rates with only an overall mean of 1.5 percent (sd = 1.42). By the end April 2021, vaccines became widely available to everyone who wants a shot, and hence, it was a critical time point that indicates the supply might no longer be an issue. Focusing on January 2022 and the most recent rates in April 2022 provides a one-year changing timeline.

### 3.2 Measures

The key predictors are diverse racial concentrations as captured by percent of the population from a specific racial group out of the total county population (see also McLaughlin et al. 2002; Ransome et al., 2016). The data come from the Census Bureau's 2019 American Community Survey (ACS). For example, Asian concentration is measured by the percent of the county population that is non-Hispanic Asian, Black concentration, Hispanic concentration, and White concentration are all measured in the same manner. I opted not to consider concentrations of other racial groups due to the low variance issue. For example, most U.S. counties have zero or close to zero percent of Native Hawaiian/Pacific Islander and American Indian/Alaska Native. Conceptualizing racial concentration as one important dimension of residential segregation, Massey and Denton (1988: 289) referred racial concentration to “relative amount of physical space occupied by a minority group in the urban environment.” Here, racial concentration is simply defined as the county-level percentage of population from a particular race. Hence, two important differences should be noted. First, rather smaller areas in an urban environment, counties are the geographic unit of analysis. County-level racial concentration captures the concentration of different racial populations in the country and relative to different counties across the country. Second, rather on the geographic size of the county's physical space/land area, racial concentration is defined based on the total population of each county. Because counties can be big, and not all the physical space is evenly occupied, county-level racial concentration may be better indicated by the percentage of a minority group in relative to the total population, rather by the amount of certain racial population to the size of the land area.

County-level social vulnerability indicators including median household income, unemployment rate, and level of education are from the U.S. Congress Joint Economic Committee's Social Capital Project, 2018(Social Capital Project, 2018), as well as two other indicators, resources constrained health system index and healthcare accessibility barriers index, are from the COVID-19 Vaccine Coverage Index launched by Surgo Ventures in February 2021 (Mishra et al., 2021). I use a principal components analysis (PCA) to combine these measures and create a county level social vulnerability index that represents the overall level of socioeconomic disadvantage of each county. The PCA is a data reduction technique that can combine different indicators based on the common variance among the measures (see also Wu et al. 2021). Higher scores indicate higher levels of vulnerability. Political ideology is captured by the political party affiliation (percent of Republicans) and the differences between Republican votes and Democratic votes during the 2020 election in each county. The data are made publicly available by McGovern et al. (2020). I also use a principal components analysis (PCA) to combine these two measures and create a county-level political conservatism index. Additional analysis using the original indicators yield similar results. Counties with higher scores mean those counties are politically more conservative.

I also include the CDC county level COVID-19 Hesitancy Data that include county-level estimates of vaccine hesitancy rates during May–June in 2021 (see more information here: Beleche et al., 2021). County-level vaccine hesitancy is measured using the percent of individuals who reported highly unwilling to get vaccinated in each county. Other county-level controls such as COVID-19 case number as of July 2021, COVID-19 cases per thousand population, median age, and percent of rural population are also available from the merged dataset. In total, the dataset includes key information for 3,089 counties across 49 states. All variables were measured at the county level. Table 1 provides descriptive statistics for all key variables in the analysis.

**Table 1:** Descriptive statistics of key variables in analysis.

Variable	Obs	Mean	Std. Dev.	Min	Max
***Vaccination rates***					
Percent fully vaccinated, January 2021	3,097	1.46	1.42	0.0	25.4
Percent fully vaccinated, April 2021	3,097	28.85	14.32	0.0	99.9
Percent fully vaccinated, January 2022	3,097	58.57	13.10	0.0	95.0
Percent fully vaccinated, April 2022	3,089	60.19	12.54	13.5	95.0
***Racial concentrations***					
Percent Asians	3,104	1.30	2.56	0.0	41.7
Percent Blacks	3,104	8.99	14.48	0.0	87.2
Percent Hispanics	3,104	9.40	13.82	0.0	99.2
Percent Whites	3,104	76.64	19.81	0.7	100.0
***Social vulnerability***					
Median household income (in 10k)	3,104	4.78	1.25	1.9	12.6
Unemployment rate	3,104	4.01	1.64	0.0	18.8
Percent of adults with BA	3,104	20.76	9.11	3.0	80.2
Resource constrained health system index	3,104	0.50	0.29	0.0	1.0
Healthcare accessibility barriers index	3,104	0.50	0.29	0.0	1.0
Social vulnerability PCF index	3,104	0.00	1.00	−4.7	2.9
***Political ideology***					
Percent of GOP	3,104	0.65	0.16	0.1	1.0
GOP/DEM votes difference (10k)	3,104	−0.18	5.42	−188.3	11.9
Political conservatism PCF index	3,104	0.00	1.00	−10.6	6.9
***Controls***					
COVID-19 vaccine hesitancy	3,104	8.61	3.23	1.9	18.2
COVID-19 cases (1k), as of July 2021	3,104	10.63	37.71	0.0	1282.4
COVID-19 cases per thousand population	3,104	1.06	0.31	0.0	6.1
Median age	3,104	41.07	5.31	21.5	66.0
Percent rural population	3,104	58.55	31.38	0.0	100.0

### 3.3 Plan of analysis

**Fig. 1:**
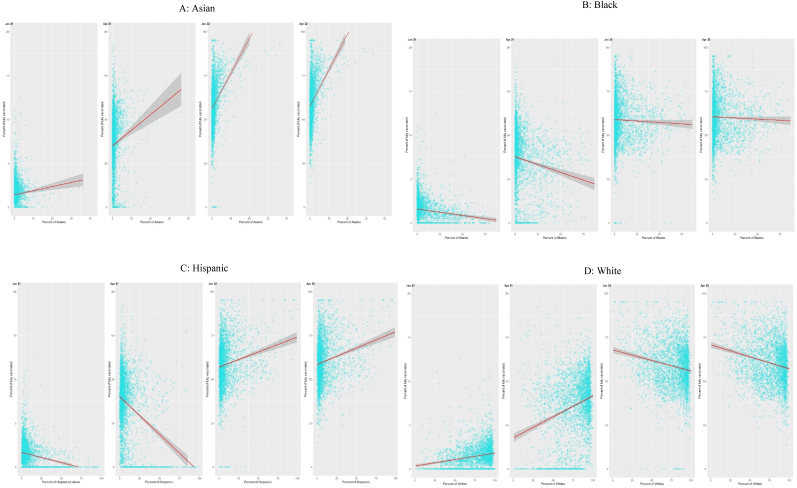
Dynamics of racial concentrations and COVID-19 vaccination rates across US counties, 2021–2022.

The analysis takes four general steps. First, I explore how concentrations of different racial groups are associated with vaccination rates over time. Specifically, I use scatterplots to visualize the associations by race categories and use the changes in Pearson correlation coefficients to indicate the changes in these associations over time. Second, to establish that social vulnerability and political ideology could be the underlying factors that connect racial concentration with vaccination rates across counties, I show that concentrations of different racial groups are associated differently with measures of social vulnerability and political ideology. Again, I use scatterplots and changes in Pearson correlation coefficients for this purpose. Third, I test whether the differential associations between racial concentration and social vulnerability and political conservatism can help explain the racially-specific and time-varying associations between racial concentration and COVID-19 vaccination rates. I use a series of mixed-effects models with counties (level one fixed effects) nested within states (level two random intercepts) to estimate the diverse effects of racial concentration on vaccination uptake by race and in different situations. Model (1), the base model, includes only concentration of a specific racial group as the main predictor with controlling county level overall COVID-19 vaccine hesitancy as well as a range of other controls such as COVID-19 cases as of July 2021, the COVID-19 rates per thousand population, median age, and percent of rural population. Model (2) adds the social vulnerability PCF index to the base model. Model (3) adds the political conservatism index to the base model. Model (4), the full model, includes all variables. The analysis is conducted separately across race categories including Asian, Black, Hispanic, as well as White as well as by different time points including January 2021, April 2021, January 2022, and April 2022. A test of multicollinearity using the variance inflating factor (VIF) analysis after regressions shows that all variables included have a VIF score lower than 3 (VIF<10, acceptable). Comparing the changes in the size and directions of the effects of racial concentration, and the effects of two mechanism variables social vulnerability and political ideology provides a way to illustrate not only how the association between racial concentration and vaccination differs across race and over time, but also the unequal and changing roles both social vulnerability and political ideology play in creating the racially specific and time-varying patterns. Finally, I report results from mediation analysis and show that social vulnerability and political ideology significantly mediate the associations between racial concentration and vaccination rates over time and across race groups (see Fig. 1, Fig. 4A, Fig. 4B).

## 4 Empirical findings

**Fig. 2:**
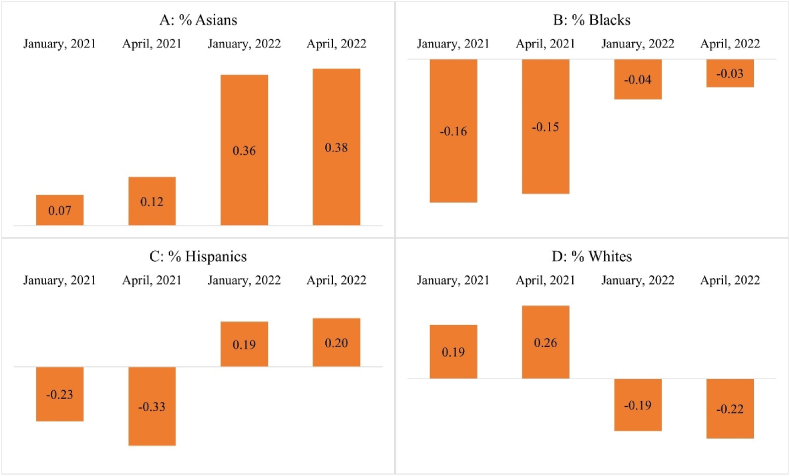
Changes in the Pearson correlation coefficients between vaccination rates and racial concentration of Asians (A), Blacks (B), Hispanics (C), and Whites (D) over four different time points.

First, I consider how concentrations of different racial groups are associated with vaccination rates over time. Fig. 1 visualizes the scatterplots between county-level concentrations of Asians (A), Blacks (B), Hispanics (C), and Whites (D) and vaccination rates at four different times from early vaccine rollout to the time when COVID-19 vaccines become widely available (January 2021, April 2021, January 2022, and April 2022). Fig. 2 plots the changes in the Pearson correlation coefficients.

Panel A shows that Asian concentration is positively associated with COVID-19 vaccination across US counties. Counties with higher proportions of Asian population show higher COVID-19 vaccination rates during early rollout in January 2021. This positive association has become increasingly stronger over time. The Pearson correlation coefficient was 0.07 in January, but it increased to 0.12 in April 2021 and further to 0.36 in January 2022 and 0.38 in April 2022.

Panel B shows that the association between Black concentration and COVID-19 vaccination has been largely negative. Counties with higher proportions of Blacks show lower COVID-19 vaccination rates. This is especially true during early rollout in January 2021 (r = −0.16) and before the COVID-19 vaccines became widely available to everyone in April 2021 (r = −0.15). The negative association became very weak in January 2022 (−0.04) and after (r = −0.03 in April 2022).

Panel C shows that the association between Hispanic concentration and COVID-19 vaccination has changed from highly negative to highly positive. During early rollout in January 2021 (r = −0.23) and before the COVID-19 vaccines became widely available to everyone in April 2021(r = −0.33), counties with higher proportions of Hispanics show lower COVID-19 vaccination rates. The opposite is true for January 2022 (r = 0.19) and April 2022 (r = 0.20).

Panel D shows that the association between White concentration and COVID-19 vaccination has changed from highly positive to highly negative. This is in sharp contrast to Hispanic concentration. Counties with higher proportions of Whites show lower higher COVID-19 vaccination rates during early rollout in January 2021 (r = 0.19) and before the COVID-19 vaccines became widely available to everyone in April 2021 (r = 0.26), but the association has become negative since January 2022 (r = −0.19).

Clearly, not only can the association between racial concentration and COVID-19 vaccination differ across racial groups, but it is also highly variable over time within each race group. The overall patterns are consistent when I visualize the associations by US states (see Appendix Fig, A1).

**Fig. 3:**
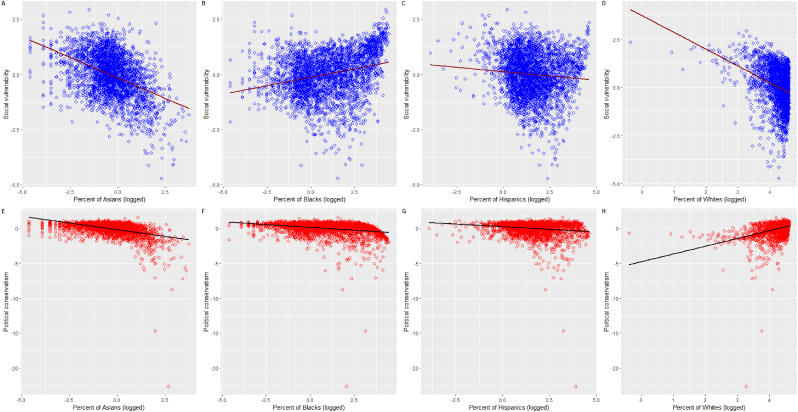
Scatterplots between social vulnerability, political conservatism, and concentrations of Asians, Blacks, Hispanics, and Whites.

Second, I consider how concentrations of different racial groups are associated with social vulnerability and political ideology. Fig. 3 shows that only Black concentration is positively associated with social vulnerability (r = 0.4). Asian concentration is negatively associated with social vulnerability (r = −0.39), a pattern that resembles White concentration (r = −0.32). Hispanic concentration is only weakly related to social vulnerability (r = 0.07). When it comes to political ideology, only White concentration is positively associated with political conservatism (r = 0.44). Asian concentration (r = −0.54), Black concentration (r = −0.32), and Hispanic concentration (r = −15) all show a negative relationship with political conservatism. These patterns demonstrate that concentrations of different racial groups are differentially associated with structural factors that matter for COVID-19 vaccination rates.

Finally, I consider how social vulnerability and political ideology may play differential roles for different race groups and at different points in time in affecting vaccination rates. Table 2A, Table 2B, Table 2C, Table 2DA–2D report results from a series of mixed-effects models with counties (level one fixed effects) nested within US states (level two random intercepts) estimating the diverse effects of racial concentration on vaccination uptake by race and in different situations. Table 2A reports the results using vaccination rates data in January 2021, Table 2B using data in April 2021, Table 2C using data in April 2021, and Table 2D using data in April 2021. Before getting into the main results, I also note that county level vaccine hesitancy does show strong negative impacts across most models, but it cannot fully explain the effect of racial concentration on vaccination rates. To facilitate the interpretation, Table 3 provides a summary of the main results across models by race categories and time points.

**Table 2A:** Mixed-effects models estimating effects of racial concentration on COVID-19 vaccination by race, January 2021

	Asian				Black				Hispanic				White			
*Predictors*	Model (1)	Model (2)	Model (3)	Model (4)	Model (1)	Model (2)	Model (3)	Model (4)	Model (1)	Model (2)	Model (3)	Model (4)	Model (1)	Model (2)	Model (3)	Model (4)
(Intercept)	1.90 ***	1.61 ***	1.56 ***	1.11 ***	2.18 ***	1.73 ***	1.50 ***	1.08 ***	2.59 ***	2.16 ***	2.16 ***	1.59 ***	1.97 ***	1.61 ***	0.95 **	0.74 *
**Racial concentration**																
% racial population	0.02	0.01	0.01	−0.01	−0.01 ***	0.01	−0.02 ***	−0.01 ***	−0.01 ***	−0.01 ***	−0.02 ***	−0.01 ***	0.00 *	0.01	0.01 ***	0.01 ***
**Mechanisms**																
Social vulnerability PCF index		−0.16 ***		−0.20 ***		−0.14 ***		−0.14 ***		−0.12 ***		−0.15 ***		−0.17 ***		−0.14 ***
Political ideology PCF index			−0.13 ***	−0.17 ***			−0.26 ***	−0.26 ***			−0.14 ***	−0.17 ***			−0.26 ***	−0.24 ***
**Controls**																
% who hesitant	−0.03	0.01	−0.01	0.04	−0.03	0.01	0.02	0.05 *	−0.06 **	−0.02	−0.03	0.01	−0.04	0.01	0.00	0.04
COVID-19 cases (1k), July 2021	0.01	0.01	0.01	0.01	0.01	0.01	−0.00 *	−0.00 **	0.01	0.01	0.01	0.01	0.01	0.01	0.01	−0.00 *
Median age	−0.01	−0.01	0.00	−0.01	−0.01	−0.01	0.00	0.00	−0.01 **	−0.01 **	−0.01 *	−0.01 *	−0.01	−0.01	−0.01 *	−0.01
COVID-19 cases/population	0.40 ***	0.42 ***	0.42 ***	0.46 ***	0.37 ***	0.41 ***	0.41 ***	0.45 ***	0.49 ***	0.50 ***	0.53 ***	0.55 ***	0.40 ***	0.42 ***	0.50 ***	0.51 ***
% rural population	−0.01 ***	−0.00 ***	−0.00 ***	−0.00 **	−0.01 ***	−0.00 ***	−0.00 ***	−0.00 **	−0.01 ***	−0.00 ***	−0.00 ***	−0.00 **	−0.01 ***	−0.00 ***	−0.00 ***	−0.00 **
**Random Effects**																
σ^2^	1.31	1.3	1.3	1.29	1.31	1.3	1.29	1.28	1.29	1.29	1.29	1.28	1.31	1.3	1.29	1.29
τ_00_	0.54 _state_	0.48 _state_	0.53 _state_	0.47 _state_	0.52 _state_	0.48 _state_	0.46 _state_	0.43 _state_	0.51 _state_	0.46 _state_	0.49 _state_	0.44 _state_	0.53 _state_	0.49 _state_	0.45 _state_	0.43 _state_
ICC	0.29	0.27	0.29	0.27	0.29	0.27	0.26	0.25	0.28	0.26	0.28	0.26	0.29	0.27	0.26	0.25
N	49 _state_	49 _state_	49 _state_	49 _state_	49 _state_	49 _state_	49 _state_	49 _state_	49 _state_	49 _state_	49 _state_	49 _state_	49 _state_	49 _state_	49 _state_	49 _state_
Observations	3097	3097	3097	3097	3097	3097	3097	3097	3097	3097	3097	3097	3097	3097	3097	3097
Marginal R^2^/Conditional R^2^	0.038/0.320	0.042/0.301	0.040/0.317	0.053 /0.304	0.044/0.318	0.044/0.300	0.059/0.307	0.068/0.303	0.058/0.326	0.056/0.304	0.063/0.323	0.069/0.307	0.040/0.317	0.042/0.302	0.056/0.300	0.061/0.296

*p < 0.05 **p < 0.01 ***p < 0.001.

**Table 2B:** Mixed-effects models estimating effects of racial concentration on COVID-19 vaccination by race, April 2021

	Asian				Black				Hispanic				White			
*Predictors*	Model (1)	Model (2)	Model (3)	Model (4)	Model (1)	Model (2)	Model (3)	Model (4)	Model (1)	Model (2)	Model (3)	Model (4)	Model (1)	Model (2)	Model (3)	Model (4)
(Intercept)	32.23 ***	28.04 ***	22.71 ***	14.60 ***	36.07 ***	27.29 ***	23.64 ***	14.73 ***	38.33 ***	30.52 ***	27.20 ***	15.18 ***	38.22 ***	29.09 ***	21.20 ***	14.75 ***
**Racial concentration**																
% racial population	0.43 ***	0.26 **	0.26 ***	−0.05	0.03 *	0.08 ***	−0.13 ***	−0.09 ***	−0.05 **	−0.01	−0.06 ***	−0.02	−0.07 ***	−0.14 ***	0.06 ***	−0.01
**Mechanisms**																
Social vulnerability PCF index		−1.67 ***		−2.65 ***		−2.24 ***		−2.30 ***		−1.83 ***		−2.55 ***		−3.10 ***		−2.70 ***
Political ideology PCF index			−3.34 ***	−3.93 ***			−4.50 ***	−4.54 ***			−3.48 ***	−3.91 ***			−4.10 ***	−3.78 ***
**Controls**																
% who hesitant	−1.35 ***	−0.80 ***	−0.83 ***	0.11	−1.59 ***	−0.76 ***	−0.68 ***	0.16	−1.66 ***	−0.90 ***	−1.05 ***	0.06	−1.52 ***	−0.28	−0.88 ***	0.13
COVID-19 cases (1k), July 2021	0.01	0.01	−0.02 ***	−0.02 ***	0.01	0.001	−0.02 ***	−0.03 ***	0.00	0.00	−0.02 ***	−0.02 ***	0.00	−0.01	−0.02 ***	−0.02 ***
Median age	0.22 ***	0.20 ***	0.30 ***	0.27 ***	0.20 ***	0.19 ***	0.28 ***	0.27 ***	0.17 ***	0.17 ***	0.25 ***	0.26 ***	0.27 ***	0.33 ***	0.23 ***	0.28 ***
COVID-19 cases/population	2.95 ***	3.17 ***	3.59 ***	4.06 ***	2.69 ***	3.26 ***	3.38 ***	3.98 ***	2.97 ***	3.11 ***	3.89 ***	4.20 ***	2.31 ***	2.61 ***	3.86 ***	4.01 ***
% rural population	−0.03 ***	−0.02 ***	−0.01	0.01	−0.04 ***	−0.02 **	0.00	0.01	−0.04 ***	−0.03 ***	−0.01	0.01	−0.03 ***	−0.01 *	0.00	0.01
**Random Effects**																
σ^2^	56.29	55.42	51.8	49.51	56.69	55.1	50.85	49.08	56.63	55.57	51.69	49.5	56.09	53.27	51.64	49.5
τ_00_	103.92 _state_	88.92 _state_	92.44 _state_	75.37 _state_	110.84 _state_	89.35 _state_	86.40 _state_	72.98 _state_	109.89 _state_	89.80 _state_	94.78 _state_	75.33 _state_	112.18 _state_	87.60 _state_	90.52 _state_	75.77 _state_
ICC	0.65	0.62	0.64	0.6	0.66	0.62	0.63	0.6	0.66	0.62	0.65	0.6	0.67	0.62	0.64	0.6
N	49 _state_	49 _state_	49 _state_	49 _state_	49 _state_	49 _state_	49 _state_	49 _state_	49 _state_	49 _state_	49 _state_	49 _state_	49 _state_	49 _state_	49 _state_	49 _state_
Observations	3097	3097	3097	3097	3097	3097	3097	3097	3097	3097	3097	3097	3097	3097	3097	3097
Marginal R^2^/Conditional R^2^	0.135/0.696	0.106/0.657	0.141/0.691	0.135/0.657	0.148/0.712	0.097/0.656	0.162/0.689	0.150/0.658	0.156/0.713	0.112/0.661	0.158/0.703	0.137/0.658	0.146/0.715	0.092/0.657	0.155/0.693	0.132/0.657

*p<0.05 **p<0.01 ***p<0.001.

**Table 2C:** Mixed-effects models estimating effects of racial concentration on COVID-19 vaccination by race, January 2022

	Asian				Black				Hispanic				White			
*Predictors*	Model (1)	Model (2)	Model (3)	Model (4)	Model (1)	Model (2)	Model (3)	Model (4)	Model (1)	Model (2)	Model (3)	Model (4)	Model (1)	Model (2)	Model (3)	Model (4)
(Intercept)	76.21 ***	70.70 ***	58.17 ***	48.67 ***	84.83 ***	71.32 ***	60.73 ***	49.96 ***	80.17 ***	66.13 ***	59.24 ***	41.34 ***	91.50 ***	76.58 ***	63.54 ***	54.87 ***
**Racial concentration**																
% racial population	0.97 ***	0.73 ***	0.64 ***	0.15	0.07 ***	0.14 ***	−0.23 ***	−0.16 ***	0.15 ***	0.21 ***	0.12 ***	0.20 ***	−0.20 ***	−0.33 ***	0	−0.14 ***
**Mechanisms**																
Social vulnerability PCF index		−2.28 ***		−3.92 ***		−3.53 ***		−3.49 ***		−3.48 ***		−4.61 ***		−5.67 ***		−4.98 ***
Political ideology PCF index			−6.34 ***	−7.17 ***			−8.43 ***	−8.40 ***			−6.48 ***	−7.19 ***			−6.56 ***	−5.89 ***
**Controls**																
% who hesitant	−2.37 ***	−1.64 ***	−1.38 ***	−0.26	−2.93 ***	−1.64 ***	−1.11 ***	−0.15	−2.61 ***	−1.25 ***	−1.44 ***	0.14	−2.76 ***	−0.71 ***	−1.63 ***	−0.13
COVID-19 cases (1k), July 2021	0.01 **	0.02 **	−0.02 ***	−0.02 ***	0.03 ***	0.02 ***	−0.02 ***	−0.03 ***	0.02 ***	0.02 ***	−0.02 **	−0.02 ***	0.02 **	0.01	−0.01 **	−0.02 ***
Median age	0.09	0.05	0.23 ***	0.17 ***	0.04	0.02	0.18 ***	0.16 ***	0.10 *	0.11 *	0.25 ***	0.26 ***	0.26 ***	0.36 ***	0.19 ***	0.28 ***
COVID-19 cases/population	3.85 ***	4.10 ***	5.09 ***	5.76 ***	3.31 ***	4.14 ***	4.62 ***	5.53 ***	2.05 **	2.28 **	3.77 ***	4.40 ***	2.20 **	2.74 ***	4.66 ***	4.93 ***
% rural population	−0.08 ***	−0.07 ***	−0.03 ***	−0.01	−0.09 ***	−0.07 ***	−0.04 ***	−0.01	−0.09 ***	−0.07 ***	−0.04 ***	0.00	−0.09 ***	−0.05 ***	−0.04 ***	−0.01
σ^2^	101.22	99.82	85.13	79.95	103.14	99.44	82.93	78.56	102.19	98.36	85.25	77.4	97.23	87.46	86.2	78.35
τ_00_	67.05 _state_	47.04 _state_	41.02 _state_	25.97 _state_	83.09 _state_	46.87 _state_	32.34 _state_	23.54 _state_	74.98 _state_	41.76 _state_	41.53 _state_	24.38 _state_	85.02 _state_	44.21 _state_	44.37 _state_	28.11 _state_
ICC	0.4	0.32	0.33	0.25	0.45	0.32	0.28	0.23	0.42	0.3	0.33	0.24	0.47	0.34	0.34	0.26
N	49 _state_	49 _state_	49 _state_	49 _state_	49 _state_	49 _state_	49 _state_	49 _state_	49 _state_	49 _state_	49 _state_	49 _state_	49 _state_	49 _state_	49 _state_	49 _state_
Observations	3097	3097	3097	3097	3097	3097	3097	3097	3097	3097	3097	3097	3097	3097	3097	3097
Marginal R^2^/Conditional R^2^	0.364/0.617	0.337/0.549	0.422/0.610	0.434/0.573	0.376/0.655	0.324/0.541	0.458/0.610	0.462/0.586	0.383/0.644	0.344/0.540	0.428/0.615	0.441/0.575	0.397/0.678	0.364/0.577	0.426/0.621	0.426/0.578

*p<0.05 **p<0.01 ***p<0.001.

**Table 2D:** Mixed-effects models estimating effects of racial concentration on COVID-19 vaccination by race, April 2022

	Asian				Black				Hispanic				White			
*Predictors*	Model (1)	Model (2)	Model (3)	Model (4)	Model (1)	Model (2)	Model (3)	Model (4)	Model (1)	Model (2)	Model (3)	Model (4)	Model (1)	Model (2)	Model (3)	Model (4)
(Intercept)	78.58 ***	73.72 ***	60.29 ***	51.20 ***	84.57 ***	72.75 ***	60.89 ***	51.28 ***	80.72 ***	68.65 ***	59.74 ***	43.62 ***	91.56 ***	77.76 ***	64.00 ***	55.93 ***
**Racial concentration**																
% racial population	0.72 ***	0.51 ***	0.37 ***	−0.09	0.08 ***	0.15 ***	−0.22 ***	−0.15 ***	0.13 ***	0.18 ***	0.10 ***	0.17 ***	−0.21 ***	−0.33 ***	−0.01	−0.13 ***
**Mechanisms**																
Social vulnerability PCF index		−1.99 ***		−3.69 ***		−3.09 ***		−3.07 ***		−2.95 ***		−4.09 ***		−5.20 ***		−4.51 ***
Political ideology PCF index			−6.51 ***	−7.30 ***			−8.39 ***	−8.37 ***			−6.57 ***	−7.20 ***			−6.54 ***	−5.93 ***
**Controls**																
% who hesitant	−2.45 ***	−1.81 ***	−1.45 ***	−0.38 *	−2.91 ***	−1.78 ***	−1.13 ***	−0.27	−2.60 ***	−1.42 ***	−1.44 ***	−0.01	−2.71 ***	−0.81 ***	−1.62 ***	−0.24
COVID-19 cases (1k), July 2021	0.01 *	0.01 *	−0.03 ***	−0.03 ***	0.02 ***	0.02 **	−0.03 ***	−0.03 ***	0.02 ***	0.02 **	−0.02 ***	−0.03 ***	0.01	0	−0.02 ***	−0.03 ***
Median age	0.12 **	0.09 *	0.26 ***	0.21 ***	0.09 *	0.07	0.23 ***	0.21 ***	0.14 **	0.14 ***	0.29 ***	0.30 ***	0.31 ***	0.41 ***	0.24 ***	0.33 ***
COVID-19 cases/population	3.80 ***	4.02 ***	5.04 ***	5.67 ***	3.46 ***	4.18 ***	4.74 ***	5.55 ***	2.30 ***	2.49 ***	4.04 ***	4.58 ***	2.29 ***	2.79 ***	4.73 ***	4.97 ***
% rural population	−0.09 ***	−0.08 ***	−0.04 ***	−0.02 **	−0.10 ***	−0.08 ***	−0.04 ***	−0.02 **	−0.10 ***	−0.09 ***	−0.05 ***	−0.02 *	−0.10 ***	−0.06 ***	−0.05 ***	−0.02 **
**Random Effects**																
σ^2^	85.99	84.93	68.93	64.31	86.52	83.69	66.35	62.97	86.13	83.45	68.6	62.43	80.36	72.12	69.23	62.81
τ_00_	61.93 _state_	45.03 _state_	36.76 _state_	24.37 _state_	76.14 _state_	46.09 _state_	28.54 _state_	21.77 _state_	66.98 _state_	39.19 _state_	35.96 _state_	21.52 _state_	75.45 _state_	39.96 _state_	39.75 _state_	25.49 _state_
ICC	0.42	0.35	0.35	0.27	0.47	0.36	0.3	0.26	0.44	0.32	0.34	0.26	0.48	0.36	0.36	0.29
N	49 _state_	49 _state_	49 _state_	49 _state_	49 _state_	49 _state_	49 _state_	49 _state_	49 _state_	49 _state_	49 _state_	49 _state_	49 _state_	49 _state_	49 _state_	49 _state_
Observations	3089	3089	3089	3089	3089	3089	3089	3089	3089	3089	3089	3089	3089	3089	3089	3089
Marginal R^2^/Conditional R^2^	0.397/0.650	0.372/0.590	0.469/0.654	0.478/0.621	0.406/0.684	0.358/0.586	0.506/0.655	0.506/0.633	0.413/0.670	0.378/0.577	0.474/0.655	0.485/0.617	0.431/0.707	0.401/0.615	0.471/0.664	0.470/0.623
*p<0.05 **p<0.01 ***p<0.001

**Table 3:** Summary of model results.

Racial concentration	Mechanisms	Time			
		Jan-21^a^	Apr-22	Jan-22	Apr-22
**Asian**	Model (1): base	0.02	0.43 ***	0.97 ***	0.72 ***
	Model (2): social vulnerability	0.00	0.26 **	0.73 ***	0.51 ***
	Model (3): political conservatism	0.01	0.26 ***	0.64 ***	0.37 ***
	Model (4): full	−0.01	−0.05	0.15	−0.09

**Black**	Model (1): base	−0.01 ***	0.03 *	0.07 ***	0.08 ***
	Model (2): social vulnerability	0.00	0.08 ***	0.14 ***	0.15 ***
	Model (3): political conservatism	−0.02 ***	−0.13 ***	−0.23 ***	−0.22 ***
	Model (4): full	−0.01 ***	−0.09 ***	−0.16 ***	−0.15 ***

**Hispanic**	Model (1): base	−0.01 ***	−0.05 **	0.15 ***	0.13 ***
	Model (2): social vulnerability	−0.01 ***	−0.01	0.21 ***	0.18 ***
	Model (3): political conservatism	−0.02 ***	−0.06 ***	0.12 ***	0.10 ***
	Model (4): full	−0.01 ***	−0.02	0.20 ***	0.17 ***

**White**	Model (1): base	0.00 *	−0.07 ***	−0.20 ***	−0.21 ***
	Model (2): social vulnerability	0.00	−0.14 ***	−0.33 ***	−0.33 ***
	Model (3): political conservatism	0.01 ***	0.06 ***	0.00	−0.01
	Model (4): full	0.01 ***	−0.01	−0.14 ***	−0.13 ***

a Coefficients for January 2021 are less interpretable given the low vaccination rates across all counties during the early rollout period (mean = 1.5, sd = 1.4).

Asian: Model (1)s confirm that higher Asian concentration is positively associated with vaccination uptake and the positive association becomes increasingly stronger over time. Model (2)s show that social vulnerability (counties with more Asians are lower in social vulnerability index) partially explains the positive effect. The effect of Asian concentration becomes smaller when social vulnerability index is included. This is consistent over time. Model (3)s show that political ideology can also help explain partially the effect of Asian concentration. When both social vulnerability and political ideology are considered, Model (4)s show that the effect of Asian concentration on vaccination turns becomes insignificant. These results show that the association between Asian concentration and vaccination uptake across US counties could be driven by both social vulnerability and political ideology.

Black: Model (1)s shows that higher Black concentration is also negatively associated with vaccination uptake in January 2021 during the early rollout period, but it has become positive in April 2021 and after. The size of the positive effect has also increased. Model (2)s show that when social vulnerability is considered, the negative effect of Black concentration disappears for January 2021, and since April 2021, the positive effect of Black concentration has increased even more in size. This means that social vulnerability (counties with more Blacks are higher in social vulnerability index) plays an essential role in shaping the Black concentration and COVID-19 vaccination association. When including political conservatism index, Model (3)s show that the effect of Black concentration becomes highly negative and also more substantial in terms of the effect size. The pattern seems to be stable when both social vulnerability and political ideology are both included and over time as shown in Model (4)s. These results also show that the association between Black concentration and vaccination uptake across US counties is influenced by both political ideology and social vulnerability.

Hispanic: Model (1)s show that higher Hispanic concentration is negatively associated with vaccination uptake in January and April 2021, but the association has become positive since January 2022. Model (2)s show that when social vulnerability is considered, the effect of Hispanic concentration becomes insignificant in January 2021, but all significant and positive after that. Adding political ideology, Model (3)s shows that the negative effect of Hispanic concentration become larger in size in January 2021 and April 2021. The smaller positive effect in January 2022 and April 2022 indicates that that lower political conservatism among Latino communities may help compress the effect of Hispanic concentration on COVID-19 vaccination.

White: Model (1)s show that higher White concentration is positively associated with vaccination uptake in January 2021, but the association has become negative since April 2021. Model (2)s shows that the negative effect becomes larger in size when social vulnerability is included. This means that if it were not the fact that White concentration is associated with higher concentration of social advantage, concentration of Whites may produce an even stronger negative impact on vaccination. When political ideology being considered, Model (3)s show that the negative effect of White concentration becomes positive or disappear. All factors being considered, Model (4)s show that White concentration is positively associated with vaccination uptake early one, but the association has become negative since January 2022.

Finally, to provide further support that both social vulnerability and political conservatism significantly mediate the associations between racial concentration and vaccination rates over time as well as across race categories, Fig. 4A & B report results from formal mediation analysis. To simplify the analysis, I focus on only two time points (April 2021 and April 2022). Fig. 4A reports the results when social vulnerability is the mediator. The highly significant Average Causal Mediation Effects (ACME) illustrate that social vulnerability significantly mediates the associations between racial concentration and vaccination rates across race groups and at both time points. However, the ACME of social vulnerability are positive for Asians and Whites due to their lower scores but negative for Blacks and Hispanics because of their higher scores on social vulnerability.

**Fig. 4A:**
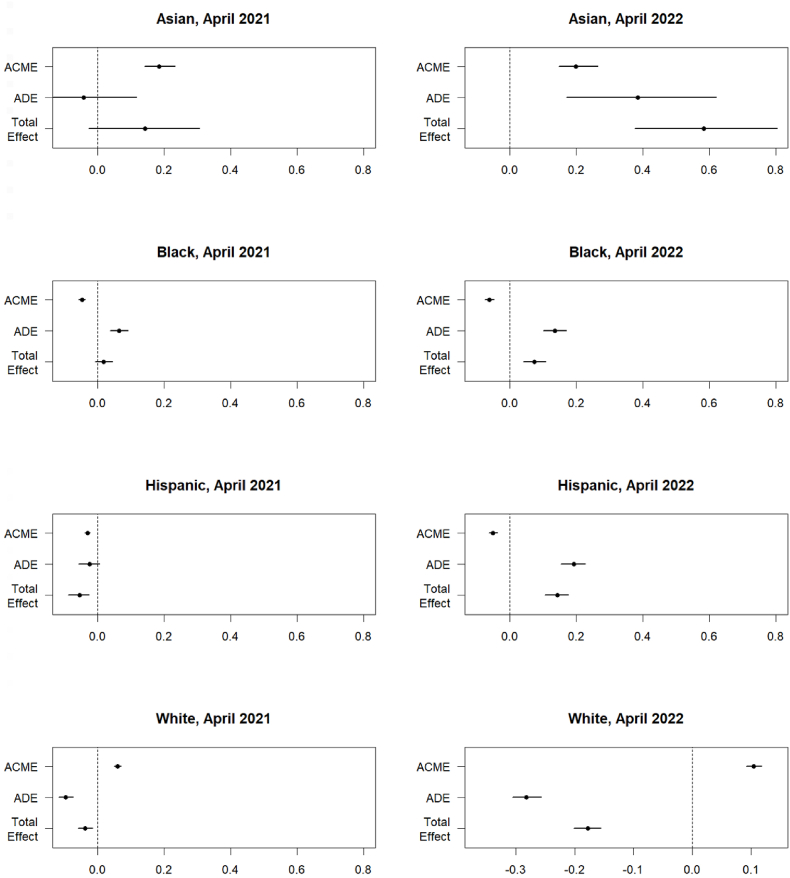
Mediation effects of social vulnerability on the associations between racial concentration and vaccination rates across race categories and over time.

**Fig. 4B:**
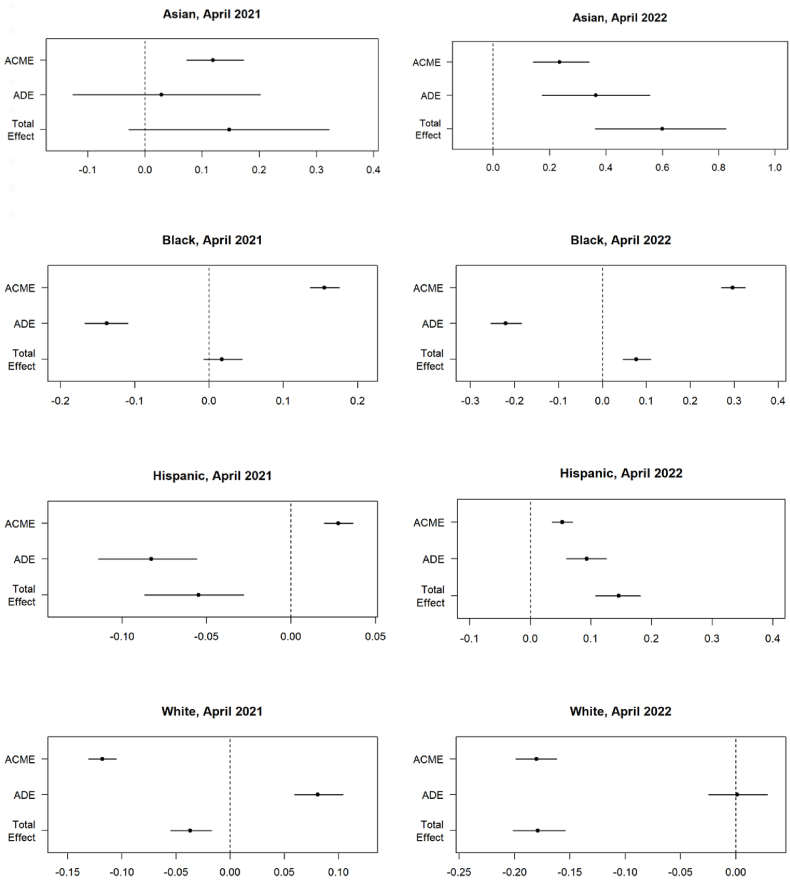
Mediation effects of political conservatism on the associations between racial concentration and vaccination rates across race categories and over time.

Fig. 4B reports the results when political conservatism is the mediator. Clearly, political conservatism also significantly mediates the associations between racial concentration and vaccination across race groups and at both time points as seen from the highly significant Average Causal Mediation Effects (ACME). Across racial minority groups, political conservatism shows positive mediation effects due to their relative lower levels of political conservatism, while for Whites, it has negative mediation effects because of the higher levels of political conservatism.

## 5 Discussion

Data from the CDC show that, as of April 27, 2022, only about 66 percent of the US population are fully vaccinated (76% among the >18 years of age). However, the vaccination rates vary substantially and also rise unevenly across geographic areas, ranging from less than 15 percent in some counties to more than 95 percent in others (see Table 1). Given that COVID-19 vaccines have become widely available since April 2021 and they are also free to everyone, why geographic variations in vaccination continue to be substantial is an important question to ask. In this article, I have considered how racial concentration affects variations in COVID-19 vaccination. The general goal is to demonstrate first the association between racial concentration and vaccination uptake differ by race group and over time, and second the racially specific and time-varying patterns are tied to the fact that concentrations of different racial and ethnic groups are differentially associated with social vulnerability and political conservatism. I have pursued the goal by simply comparing changes in regression coefficients across different models. I have also used formal mediation analyses to further confirm their significant roles in shaping the patterns of racial concentration and vaccination uptake across race and over time.

While existing studies have shown that concentration of racial minorities is associated with lower levels of vaccine uptake especially during early rollout when the supply of COVID-19 vaccines was limited (McFadden et al., 2021; Nguyen et al., 2022; DiRago 2022), this study helps advance current knowledge in several major ways. First, I have shown that concentrations of racial minorities are not always associated with lower COVID-19 vaccine uptake. The association between racial concentration and COVID-19 vaccination differs across race categories and over time. Specifically, Asian concentration is positively associated with COVID-19 vaccination, and the association has become increasingly stronger over time. The association between Black concentration and COVID-19 vaccination is largely negative but it has become weaker over time. The association between Hispanic concentration and COVID-19 vaccination has changed from highly negative during early vaccination rollout to highly positive after the vaccines became widely available.

Second, besides concentrations of racial minorities, I have also considered White concentration and how it affects COVID-19 vaccination rates. I show that the association between White concentration and COVID-19 vaccination was positive during early rollout. This is largely due to the fact that White concentration means concentration of social privileges. However, the association has become negative as the rates among racialized communities increased over time. The slower growth in vaccination rates among White communities over time reflects the fact that White concentration is associated with higher levels of political conservatism.

## 6 Conclusion

Combining data from multiple sources including the U.S. Centers for Disease Control and Prevention's real-time data on vaccinations rates, in this article I have explored the diverse and changing effects of racial concentration (% of county population being Black, Hispanic, Asian, and White) on COVID-19 vaccination uptake at four different time points over the course of the COVID-19 vaccination rollout. The findings of this study provide further support that vaccine hesitancy should not be taken as the root cause for the lower vaccination rates often observed among racial minorities. I first made this argument from a comprehensive literature review that suggests racial minorities do not necessarily show higher vaccine hesitancy (e.g., Asians show lower), and they have also become more willing to receive vaccines over time (e.g., Blacks). To further support my argument, I move beyond individual level analysis and examine how racial concentration and vaccination are related at the place level. My analysis shows that although vaccine hesitancy does appear to have a strong negative effect on vaccination rates, racial concentration still shows strong impacts on vaccination rates across race categories while controlling for vaccine hesitancy.

The findings provide support for the fundamental cause theory that suggests socioeconomic inequality and structural racism as the root causes of racial and ethnic disparities in health outcomes (Link & Jo, 1995; 2015; Williams and Collins 2016). My analysis shows that socioeconomic disadvantage and political ideology are two major factors underlying the geographic distribution of vaccination rates across counties with varying levels of concentrations of different racial groups. I have also shown that social vulnerability and political ideology are differentially associated with concentrations of different racial populations and over time, thereby creating racially specific and time-varying patterns of racial concentrations and COVID-19 vaccinations in the United States. For example, during the early rollout period, Black concentration has a negative impact on vaccination uptake is from its association with higher concentration of disadvantage that affects the access to vaccines. Whites are more likely to be republicans and are more likely to live in counties with higher levels of political conservatism. Hence, White concentration has a negative impact on vaccination uptake from more recent data after political ideology, rather the supply of vaccines, has become the dominant force in shaping people's willingness to get themselves vaccinated.

Findings of this study also suggest the need to pay attention to the particular vulnerability that members of different racial groups experience over the course of the pandemic. Acknowledging and addressing group-specific patterns and barriers for different racial groups is crucial for achieving effective and equitable responses and for reducing racial disparities during disease outbreaks.

## Funding

Funding provided by the 10.13039/501100000024Canadian Institutes of Health Research (PI: Cary Wu, FRN-170368) and The Social Sciences and Humanities Research Council (PI: Cary Wu, 435-2022-0691).

**Fig. A1:**
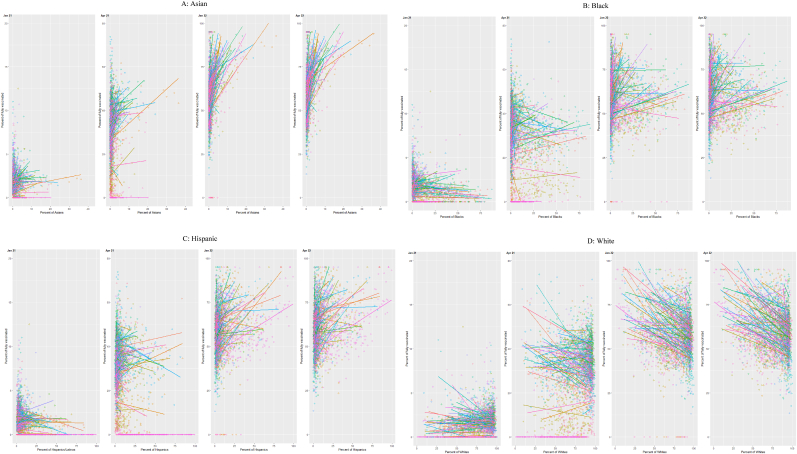
Vaccination rates and concentrations of Asians (A), Blacks (B), Hispanics (C), and Whites (D) across US counties by US states .

## Ethical statements

All data used in analysis are from publicly available sources.
